# SARS-CoV-2 Impact on Red Blood Cell Morphology

**DOI:** 10.3390/biomedicines11112902

**Published:** 2023-10-26

**Authors:** Kirill A. Kondratov, Alexander A. Artamonov, Vladimir Yu. Mikhailovskii, Anastasiya A. Velmiskina, Sergey V. Mosenko, Evgeniy A. Grigoryev, Anna Yu. Anisenkova, Yuri V. Nikitin, Svetlana V. Apalko, Natalya N. Sushentseva, Andrey M. Ivanov, Sergey G. Scherbak

**Affiliations:** 1 City Hospital No. 40, St. Petersburg 197706, Russia; 2 S. M. Kirov Military Medical Academy, St. Petersburg 194044, Russia; 3 Saint-Petersburg State University, St. Petersburg 199034, Russia

**Keywords:** COVID-19, erythrocyte, red blood cells, cytokine storm, severe COVID-19, low-voltage scanning electron microscopy, erythrocyte size

## Abstract

Severe COVID-19 alters the biochemical and morphological characteristics of blood cells in a wide variety of ways. To date, however, the vast majority of research has been devoted to the study of leukocytes, while erythrocyte morphological changes have received significantly less attention. The aim of this research was to identify erythrocyte morphology abnormalities that occur in COVID-19, compare the number of different poikilocyte types, and measure erythrocyte sizes to provide data on size dispersion. Red blood cells obtained from 6 control donors (800–2200 cells per donor) and 5 COVID-19 patients (800–1900 cells per patient) were examined using low-voltage scanning electron microscopy. We did not discover any forms of erythrocyte morphology abnormalities that would be specific to COVID-19. Among COVID-19 patients, we observed an increase in the number of acanthocytes (*p* = 0.01) and a decrease in the number of spherocytes (*p* = 0.03). In addition, our research demonstrates that COVID-19 causes an increase in the median (*p* = 0.004) and interquartile range (*p* = 0.009) when assessing erythrocyte size. The limitation of our study is a small number of participants.

## 1. Introduction

Since the beginning of the pandemic in 2020, COVID-19 has raised many questions regarding our understanding of the immunopathogenesis of viral infections. To date, there is still no common opinion that would unite and explain the changes that occur in the human body during a cytokine storm initiated by SARS-CoV-2 infection. A significant part of studies by research groups from all around the world is dedicated to researching the immune response to COVID-19 [1], often focusing on cytokines, inflammatory markers [2], and quantitative and morphological parameters of the immune system [3,4]. Fewer studies make an attempt to analyze red blood cells (RBC), specifically the morphology of erythrocytes in patients undergoing a cytokine storm. Given the fact that COVID-19 primarily affects the respiratory system [5], often inducing hypoxia [6] and resulting in conditions that a human becomes vulnerable to during low-oxygen saturation, it is probable to assume that COVID-19 can affect the main transporters of O_2_ in the human body—erythrocytes [7]. Our aim was to identify changes that occur in red blood cells during a COVID-19-induced cytokine storm, to study RBC morphology using low-voltage scanning electron microscopy (LVSEM) images, and conduct a comparative RBC morphology analysis between the healthy donors and the patients infected with SARS-CoV-2.

## 2. Materials and Methods

### 2.1. Patients and Data Collection

A total of 11 research participants were divided into 2 groups: 5 COVID-19 patients and 6 healthy donors. Our control group consisted of 6 healthy donors (mean age 51 ± 22).

The 5 COVID-19 patients (mean age 67 ± 11) presented to our hospital from 1 April 2022, to 23 August 2022. All patients were laboratory confirmed to be SARS-CoV-2 infected by real-time RT-PCR. A total of 3 patients were admitted to the intensive care unit, and 2 in the infectious disease unit. Severe patients were admitted to the ICU according to our national clinical guidelines as follows: body temperature ≥ 39 °C, respiratory rate ≥ 30/min, oxygen saturation (SpO2) ≤ 93%. The study was approved by the expert ethics council of St. Petersburg City Hospital No. 40 (protocol No. 171 of 18 May 2020).

### 2.2. Blood Sample Preparation

Fasting whole blood from every patient was collected aseptically by venipuncture into ethylenediamine tetraacetic acid (EDTA) collection tubes on the 4th day of hospital admission. Whole blood was centrifuged at 1500 g for 10 min, the supernatant was extracted after. A total of 2 µL of cell pellet was added to 5 mL of phosphate-buffered saline. It was centrifuged at 1000 g for 10 min. We then extracted the supernatant and added 500 µL of phosphate-buffered saline to the pellet. A sample preparation for LVSEM was performed according to a standardized protocol as previously described [8].

### 2.3. Low-Voltage Scanning Electron Microscopy for Erythrocyte Size Evaluation Technique Description

Further examination was carried out on a Zeiss Merlin scanning electron microscope, at 1.00 KX magnification in high resolution mode, EHT 0.400 kV. We analyzed 10 fields of view that corresponded to 10 LVSEM images.

The Feret diameter method was chosen as the main parameter for erythrocyte size evaluation.

We measured the Feret diameter of erythrocytes in ImageJ2. (Version 1.54b 8 January 2023). The scale was calibrated according to the LVSEM image micrometer ruler. The known 10 µm (micrometers) corresponds to 137.75 pixels in all images with a resolution of 3072 × 2304. Global scaling was applied. To perform a standardized count of the Feret diameter of erythrocytes of the control and COVID-19 patients, we programmed a macro to execute an automated analyzation process. As a result of the analyzed particles process and the show overlay masks function, we received a picture for every LVSEM image with contours of counted erythrocytes, separation lines of adjacent cells, counts of the number of erythrocytes, the diameter of each cell, the minimum Feret diameter, and the erythrocyte area. The values for each cell were recorded in a database table and used for further statistical analysis.

### 2.4. Erythrocyte Morphology Study

For quantifying and presenting pathologic forms of erythrocytes in charts, we introduced strict selection criteria:

− Echinocytes—presence of three or more evenly spread spike-like protrusions of plasmalemma on membrane surface with length varying from 0.5 to 2 µm with wide base; the angle between the apical part of the spike and the surface of the echinocyte membrane is usually in the range from 100 to 130 degrees. The end sections of the spikes form an acute angle.
− Acanthocytes—presence of irregularly distributed plasmalemma protrusions in the form of spines, including single ones, from 2 µm in length. The end sections of the spines end with a club-shaped extension at the apical end. The size and shape of the spines on a single acanthocyte may vary and have no strict pattern of distribution on the membrane surface.
− Stomatocytes—increased volume compared to normocytes by 20–30% and deep slit-shaped central lumen, which on the opposite side forms a semi-oval convexity with a smooth surface. The size of the central lumen depends on the degree of crenulation and can range from wide funnel-shaped to slit-shaped. Because of the strong roundness of one of the sides, they lie on their sides and are usually easily detected.
− Ovalocytes are oval or elongated erythrocytes from ovoid to bacilliform or pencil shape. The central lumen is flattened; may not be defined. The end sections of the cells are blunt, and the membrane is smooth.
− Spherocytes are erythrocytes that have lost their biconcave shape. Spherocytes are globular in shape and lack a central lumen or depression, which is most clearly visible under light microscopy.
− Schistocytes—erythrocytes are separated into fragments 2 to 3 µm in diameter. The usual round shape is absent; instead, they have a triangular or other angular morphology. Schistocytes are also classified as any degenerately altered irregularly shaped cells not conforming to other known shapes. The central lumen zone is often absent.
− Degmacytes—a bitten cell—the cell looks as if it has been bitten; has a semicircular depression on the outer side of the membrane.
− Tear cells (dacryocytes) are drop-shaped or pear-shaped erythrocytes with one large spicule with a blunt end. Cell size varies [9,10].

For counting pathologic forms of erythrocytes, we used the Cell Counter plugin for ImageJ/Fiji by Kurt de Vos [11], in which we designated eight groups of poikilocytes as mentioned above. For counting pathologic forms we used the same images that we analyzed for measuring the size of erythrocytes.

### 2.5. Statistical Analysis

Statistical analysis was performed in RStudio (version 2022.12.0 + 353.pro3). Statistical analysis for the results was executed by applying the Wilcoxon–Mann–Whitney test. A *p*-value < 0.05 was considered statistically significant. Median value, interquartile range is presented graphically. Data visualization, images, and charts were made with RStudio tidyverse, ggplot2, and sinaplot open-source packages

## 3. Results

### 3.1. Erythrocyte Morphology Study

**Figure 1:**
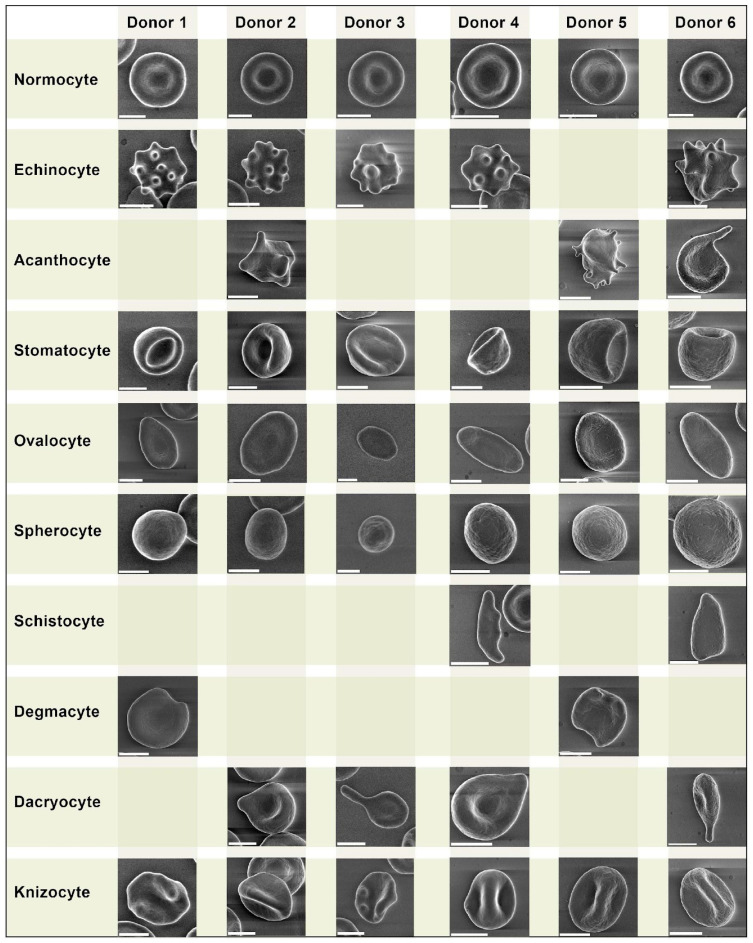
Morphological types of erythrocytes of six control donors. Images obtained by LVSEM. Scale bar is equal to 3 µm.

**Figure 2:**
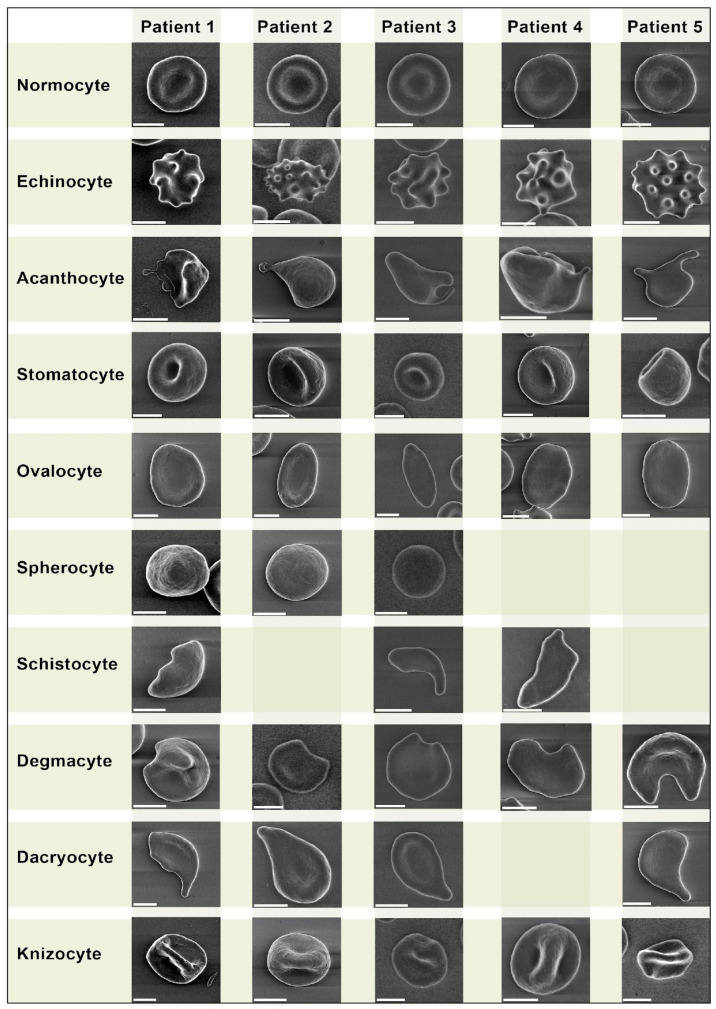
Morphological types of erythrocytes of five patients with severe COVID-19. Images obtained by LVSEM. Scale bar is equal to 3 µm.

We performed a comparative analysis of pathologic forms found in the healthy donors (Figure 1) and COVID-19 patients (Figure 2). We did not observe any erythrocyte morphology abnormalities specific to COVID-19.

Despite being similar, some pathologic forms, in particularly acanthocytes of COVID-19 patients, exhibited more pronounced plasmalemma protrusion. Acanthocytes were found in blood samples of all 5 COVID-19 patients, while in the healthy donors, they were found sporadically in only 3 samples. Overall, we did not find any significant differences in erythrocyte morphology between the healthy donors and COVID-19 patients. 

We present a comparative grid chart with images of normocytes and poikilocytes that we acquired from our blood samples. Blank grids denote cells that were not present.

### 3.2. Poikilocyte Percentage Count

**Figure 3:**
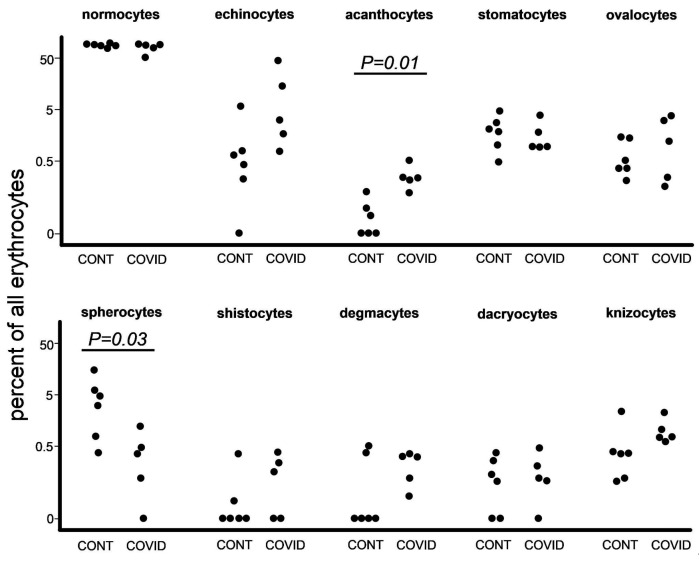
Comparison of the proportions of distinct morphological erythrocyte types (normocytes and other forms of poikilocytes) in control donors and patients with severe COVID-19.

At the next step we used the same images to count pathological forms of erythrocytes according to the criteria that we designated earlier in the Materials and Methods section (Figure 3). The difference between the overall number of poikilocytes in the healthy donors and COVID-19 patients was insignificant, 873 and 919, respectively. We found an increase in the percentage of acanthocytes among COVID-19 patients in comparison with the healthy donors. Other significant data worth noting were the raised percentages of spherocytes in the healthy donors (Tables S1 and S2).

### 3.3. Erythrocyte Size Evaluation

**Figure 4:**
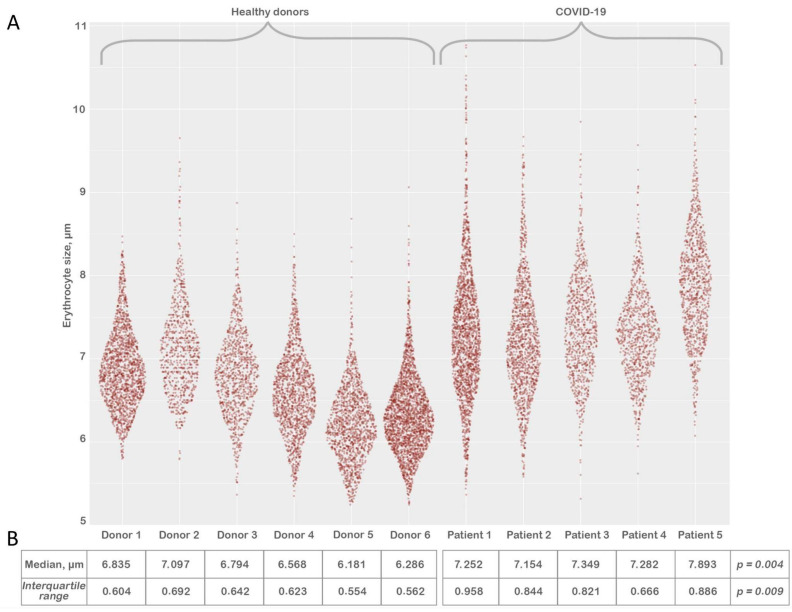
Comparison of erythrocyte sizes from six control donors and five patients with severe COVID-19 (Panel **A**). Erythrocyte size distribution (Panel **B**). Comparison of median and interquartile range of RBC size.

After processing the LVSEM images, we evaluated the size of the erythrocytes (Figure 4) by the method described above (Materials and Methods). Each sample contained between 1200 and 2270 erythrocytes. We have noted that there is a reliable increase in median (Figure 4B) and interquartile range erythrocyte size in the COVID-19 group in comparison to the healthy donors (Tables S1 and S2).

## 4. Discussion

The impact of SARS-CoV2 on red blood cells is still not clearly defined. Due to the fact that the overwhelming majority of research since the beginning of the COVID-19 pandemic is dedicated to studying immunological parameters [2,12], to date, there is little data to rely on statistically.

The significant set of features that we found during our study can potentially be reflected in a number of conditions not associated with SARS-CoV-2 infection.

We did not discover any erythrocyte abnormalities specific to COVID-19 (Figure 1 and Figure 2), similar to the mushroom-shaped cells described by Gérard et al. [13]. It should be emphasized that the vast majority of research on poikilocytosis, both in COVID-19 and in other diseases, use light microscopy to evaluate aberrant erythrocyte shapes. By employing the LVSEM method in our study, we observed precise cell morphology. However, the criteria for isolating diverse types of poikilocytes change when erythrocytes are examined using this approach. Specifically, due to the opacity of cells, the location of the cell on the substrate is crucial while monitoring erythrocytes using LVSEM. For example, a disoriented stomatocyte with its invagination pointing toward the substrate will appear as a spherocyte. Therefore, comparing our findings to that obtained from light microscopy-based examination of poikilocytes is not completely accurate. It should also be noted that patients with pre-existing diseases are significantly more likely to experience a severe course of COVID-19; therefore, any changes in erythrocyte size and the ratio of morphology deviations between the two study groups may be caused by this. None of our COVID-19 patients had any record of hematological diseases prior to the research that could directly affect the results. However, we ought to discuss the accompanying conditions of our COVID-19 patients. A total of 4 out of 5 of our COVID-19 patients had hypertensive heart disease (HHD) stage II. In their study of 102 hypertensive patients, Sileshi et al. concluded that the median values of red blood cell distribution width (RDW) in hypertensive patients were substantially higher than in healthy individuals [14]. However, RDW was within normal reference range in all of our COVID-19 patients. Two of our patients had chronic respiratory diseases—idiopathic pulmonary fibrosis and chronic bronchitis. Fois et al. note that patients with idiopathic pulmonary fibrosis demonstrate a significant increase in RDW [15], which could possibly be an indirect sign of erythrocyte size alterations; however, as mentioned earlier, in our patients, we did not observe an increase in RDW. Other accompanying diseases were: chronic hemorrhoids without exacerbation, common osteochondritis of the spine, cerebral atherosclerosis, encephalopathy grade I–II of mixed origin, history of appendectomy, chronic gastritis, without exacerbation, degenerative disc disease, secondary pulmonary hypertension, and cardiac ischemia. However, only HHD was common to all patients.

We observed a rise in acanthocyte percentage (Figure 3). The formation of acanthocytes is commonly associated with conditions such as severe liver dysfunction, neuroacanthocytosis, abetalipoproteinemia [16], malnutrition, and hypothyroidism, and post-splenectomy conditions [17]. Alterations in membrane lipids or structural proteins remain as the leading cause of acanthocyte formation [18]. Apolipoprotein A-II deficient lipoprotein accumulates in plasma as a result of liver failure, increasing cholesterol levels in RBCs. This results in abnormalities of the RBC membrane, which remodels the spleen and produces acanthocytes. A lack of lipids and vitamin E results in aberrant RBC shape in abetalipoproteinemia. [19].

It is worth noting that most of the aforementioned conditions are associated with either protein or lipid disorders, both of which are a risk factor in COVID-19 infection [20,21]. We have no exact explanation on why the percentage of spherocytes was lower among COVID-19 patients in our study (Figure 3). We can also assume that during pyrexia, cytokine storm, and general immune hyperreactivity, spherocytes undergo elimination by splenic and liver macrophages [22,23] at a more rapid rate; therefore, leading to a decrease of spherocyte presence in the peripheral blood of COVID-19 patients.

Our remarkable findings of a significant increase in erythrocyte size (Figure 4B) coincide with the studies of several research groups if extrapolated to red cell distribution width (RDW). Lippi et al. state that the absolute RDW-CV value was higher in COVID-19 patients with severe illness compared to those with mild disease [24]. Marchi et al. also confirm higher (RDW) levels in the group with elevated RBCs alterations [25]. Karampitsakos et al. state that values of RDW ≥ 14.5% were also strongly associated with an increased risk of mortality [26]. Nonetheless, it is worth noting that RBC size variations on LVSEM images are caused by variations in the cell’s diameter rather than, necessarily, by variations in the volume of the RBC measured by hematologic analyzers. Spherocytes are an example of a cell that seems smaller despite having a normal volume, while hypochromic red blood cells are thinner and spread out more in a blood smear, giving the impression that they are larger. In support of the aforementioned, Kim et al. in their research dedicated to comparing the relationship between red cell size observed through a light microscope and mean corpuscular volume (MCV) measured by an automatic hematology analyzer state that the correlation between mean corpuscular area (MCA, the size of erythrocytes observed by optical microscopy) and MCV values was poor (R = 0.641) [27]. It is worth noting that the age difference of our research participants possibly could have had an effect on the results (mean age 51 for controls and 67 for COVID-19 patients). Some alterations in the morphology and size of erythrocytes differs with age; however, according to research performed by Hoffmann et al. [28], it is noted that the most significant increase in RDW occurs in the 85+ age category. For age groups 41–55 and 56–70, they provide the following data: mean±SD RDW 12.1 ± 0.5% for females, 12.2 ± 0.6% for males, 12.3 ± 0.6 females, 12.3 ± 0.6 males, respectively. As for the MCV, the mean value for females and males in the 41–55 age group is 90.0 ± 5.1 fL and 91.2 ± 4.2 fL, respectively. For the 56–70 age group—91.1 ± 4.4 fL females, 91.4 ± 4.4 fL males. We would like to mention that routine blood tests carried out on our COVID-19 patients did not demonstrate an increasing trend in RDW or MCV. Additionally, in research performed by Zhao et al., it was stated that no significant correlations were observed between the area of RBCs and MCV. The results of correlation analysis between the area of RBCs and mean corpuscular volume (MCV), mean corpuscular hemoglobin (MCH), MCH concentration (MCHC), and red cell distribution width showed no significant correlations (p > 0.05) [29].

In order to elucidate our observations and their possible interrelations with similar findings of other research groups, we propose three possible pathogenetic pathways that could lead to the aforementioned RBC abnormalities:

Bouchla et al. [30] concluded that SARS-CoV-2 infection has an effect on RBC and that there seems to be an association between RBC markers and disease severity in their study. They report elevated hemolysis markers, specifically lactate dehydrogenase and plasma free hemoglobin. However, our patients did not exhibit any clinical or laboratory manifestations of hemolysis [31,32]. It is probable that this process was latent and could be observed only in later stages of the clinical onset. Bouchla et al. also state that COVID-19 patients’ RBCs were more sensitive to mechanical stress and exhibited significantly elevated apoptotic markers (iCa^2+^, phosphatidylserine RBC-PS) [30]. Nguyen et al. [33] confirm in their study that an increased intracellular Ca^2+^ content of RBCs results in the activation of several processes, important for phosphatidylserine exposure, eventually leading to loss of KCl and water, causing cell shrinkage, cytoskeleton destruction, membrane blebbing, and micro-vesiculation. Hoffman et al. [34] also state that the Ca^2+^-activated K^+^ channel (Gardos channel) represents the major pathway for cell shrinkage via KCl and water loss. Qadri et al. [35] and Föller et al. [36] state that cell stressors such as hypertonic shock, energy deprivation, and increased temperature may result in the activation of the aforementioned channels, which coincides with the conditions that our COVID-19 patients were experiencing. Remarkably, we found no evident signs of the aforementioned cell shrinkage. We assume that cytoskeleton destruction manifested itself in the form of poikilocytes that were almost equally presented in the healthy donors and COVID-19 patients.

Another possible pathogenetic mechanism is COVID-19-associated coagulopathy, the formation of microvascular thrombi and direct physical RBC damage. This theory is commonly spread due to the well-known pathogenetic mechanisms of SARS-CoV-2 ability to infect type II pneumocytes via angiotensin-converting enzyme 2 (ACE2) [37], cells that are in direct apposition to the alveolar vascular network leading to diffuse microvascular thrombosis [38] and high incidence of major thrombotic events in patients with COVID-19 [39]. Contrary to this data, we did not find any tracks of coagulopathy on a microscopic level such as rouleaux formations or signs of autoagglutination [39].

Plassmeyer et al. state that they observed elevated caspase-3/7 levels in red blood cells in COVID-19 patients compared to the controls [40]. It is known that the development and differentiation of erythroid progenitor cells might be regulated through caspase-dependent apoptosis [41,42]. In an ex vivo experiment, Carlile et al. demonstrated that cells that received caspase-3 siRNA during erythropoiesis were halted at the pronormoblast stage. A portion of the pronormoblasts in the siRNA-treated culture were unable to grow into basophilic normoblasts, compared to nearly all of them in the control. A total of 50% of the siRNA-treated culture remained as pronormoblasts by day 17 of the experiment [43]. Zermati et al. state that caspase inhibitors arrest erythroid development in human cells [44].

This data suggests that caspases play a key role in erythropoiesis, which leads us to a hypothesis that the elevated presence of caspase-3/7 in the RBC of COVID-19 patients [40] could be the result of immature forms entering the bloodstream. This hypothesis elucidates our findings of a significant increase in erythrocyte size. In their study, Kronstein-Wiedemann et al. [45] show that RBC precursors express ACE2 receptors on day 5 of differentiation. They used the SARS-CoV-2 alpha version (B.1.1.7) to infect CD34+ erythrocyte progenitor cells for their study. They further assessed the protein expression of key enzymes involved in heme biosynthesis. After SARS-CoV-2 infection, the expression of protoporphyrinogen-oxidase (PPOX), which catalyzes the seventh step in the biosynthesis of protoporphyrin IX, a precursor to hemoglobin, and uroporphyrinogen-decarboxylase (UROD), a homodimeric enzyme that catalyzes the fifth step in heme biosynthesis, was downregulated in CECs from all donors. In contrast to the healthy donors and recovering patients, they examined the heme and iron metabolism in blood samples from COVID-19 intensive care patients. Significant decreases in the hemoglobin content, the amounts of RBCs, the hematocrit, and peripheral lymphocyte counts in the blood samples of COVID-19 patients were observed. In comparison to the healthy donors and recovering patients, the plasma of COVID-19 patients also had abnormal iron metabolism parameters. While the inflammatory marker and iron-storage protein ferritin was strongly increased, the plasma iron and transferrin levels were decreased in COVID-19 patients [45]. SARS-CoV-2, as said earlier [37], has an increased affinity for the ACE2 receptor; therefore, making RBC precursors a direct target for viral infection, leading to significant iron dysmetabolism and disturbances of oxygen-binding capacity in severely ill COVID-19 patients [45], thereby exacerbating hypoxia. Systemic hypoxia induced by low oxygen saturation leads to an upregulated production of erythropoietin by peritubular cells of the kidney [46]. According to Bapat et al., hypoxia accelerates erythroid cell maturation and encourages erythroid differentiation by fostering the preservation of progenitor populations and promoting the production of proerythroblasts [47]. Vlaski et al. and Rogers et al. also acknowledge that low O_2_ concentration accelerates erythrocyte proliferation and differentiation [48,49].

It is well known that after each stage of cell differentiation, the erythrocyte reduces in size [50]. Given the fact that erythropoiesis takes up to 7 days [51], and that our patients’ blood samples were taken on the 4th day after hospital admission, it is possible that the significant increase in erythrocyte size is due to the prevalence of immature forms of erythrocytes in COVID-19 patients’ blood samples. This theory could also complement the findings of Plassmeyer et al. mentioned earlier [40].

## 5. Study Limitations

We analyzed a relatively small group of COVID-19 patients due to the fact that the study was conducted at a time of decline in the incidence rates of COVID-19 in St. Petersburg, Russia.

It should be mentioned that during previous waves of the epidemic, material in the form of smears and frozen blood was stored. However, these samples are not suitable for SEM studies. When blood undergoes defrosting, the morphology of red blood cells becomes altered. A standard blood smear for optical microscopy is also not suitable for SEM because the cells will be hidden by a layer of plasma proteins. Cells for SEM should first be washed with isotonic saline (PBS), fixed with glutaraldehyde, dehydrated by sequentially passing through increasing concentrations of ethanol solutions, and dried. Only then will the cell morphology be native. Such sample preparation is possible only after direct blood sampling, which cannot be completed due to the lack of patients with severe cases. We are constantly monitoring the presence of patients with cytokine storm. However, over the past 10 months, no such patients in St. Petersburg without chronic inflammatory processes have been found. Thus, a set of new samples can only be possible in the event of a new COVID-19 wave with the appearance of patients with cytokine storm.

## 6. Conclusions

Using low-voltage scanning electron microscopy, in our research, we demonstrate that severe COVID-19 causes an increase in the size and dispersion of erythrocytes, and increase in the number of acanthocytes, and a decrease in the number of spherocytes. A relatively small cohort was studied due to the low incidence rate of COVID-19 in St. Petersburg.

## Data Availability

The results of size calculations and number of different forms of erythrocytes are presented in the Supplement Materials section. All micrographs of erythrocytes obtained during the research can be provided at the first request sent to kondratovk.kirill@yandex.ru.

## Supplementary Materials

The following supporting information can be downloaded at: https://www.mdpi.com/article/10.3390/biomedicines11112902/s1, Table S1: Erythrocyte types; Table S2: Erythrocyte size.
